# V(D)J Recombination: Recent Insights in Formation of the Recombinase Complex and Recruitment of DNA Repair Machinery

**DOI:** 10.3389/fcell.2022.886718

**Published:** 2022-04-29

**Authors:** Shaun M. Christie, Carel Fijen, Eli Rothenberg

**Affiliations:** Department of Biochemistry and Molecular Pharmacology, NYU Grossman School of Medicine, New York, NY, United States

**Keywords:** V(D)J rearrangements, DNA repair, nonhomologous end joining, chromatin 3D architecture, recombinase activating gene

## Abstract

V(D)J recombination is an essential mechanism of the adaptive immune system, producing a diverse set of antigen receptors in developing lymphocytes via regulated double strand DNA break and subsequent repair. DNA cleavage is initiated by the recombinase complex, consisting of lymphocyte specific proteins RAG1 and RAG2, while the repair phase is completed by classical non-homologous end joining (NHEJ). Many of the individual steps of this process have been well described and new research has increased the scale to understand the mechanisms of initiation and intermediate stages of the pathway. In this review we discuss 1) the regulatory functions of RAGs, 2) recruitment of RAGs to the site of recombination and formation of a paired complex, 3) the transition from a post-cleavage complex containing RAGs and cleaved DNA ends to the NHEJ repair phase, and 4) the potential redundant roles of certain factors in repairing the break. Regulatory (non-core) domains of RAGs are not necessary for catalytic activity, but likely influence recruitment and stabilization through interaction with modified histones and conformational changes. To form long range paired complexes, recent studies have found evidence in support of large scale chromosomal contraction through various factors to utilize diverse gene segments. Following the paired cleavage event, four broken DNA ends must now make a regulated transition to the repair phase, which can be controlled by dynamic conformational changes and post-translational modification of the factors involved. Additionally, we examine the overlapping roles of certain NHEJ factors which allows for prevention of genomic instability due to incomplete repair in the absence of one, but are lethal in combined knockouts. To conclude, we focus on the importance of understanding the detail of these processes in regards to off-target recombination or deficiency-mediated clinical manifestations.

## Introduction

**FIGURE 1:**
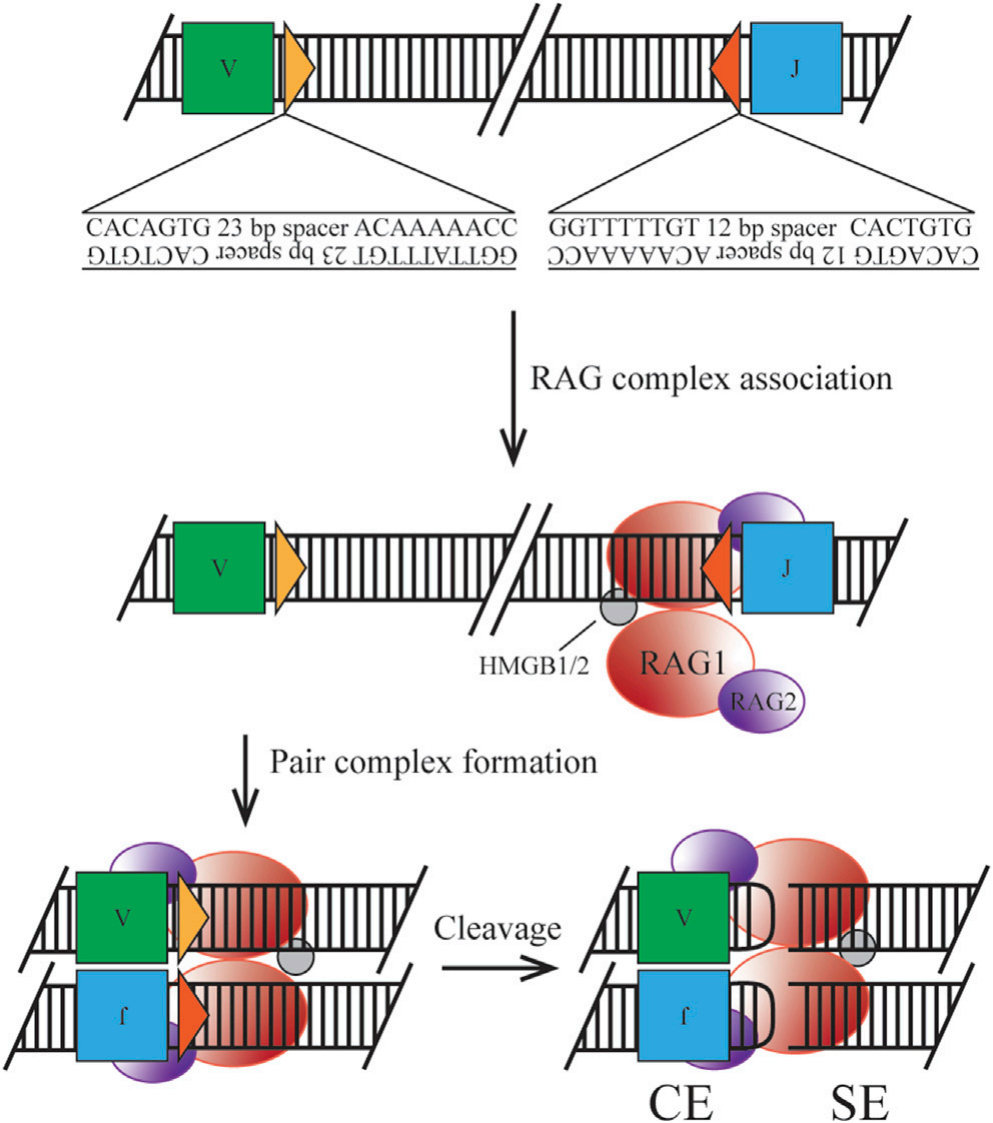
Classical mechanism of RAG initiated cleavage. Ig loci segments are flanked by 12/23 RSS containing a heptamer and nonamer sequence for RAG complex association. Synapsis of two RSS sites allows for nicking, cleavage by transesterification, and hairpin formation to form the post cleavage complex.

An essential trait of an effective adaptive immune response is the generation of a diverse set of antigen receptors. Developing lymphocytes undergo a process of regulated DNA cleavage and subsequent repair, termed V(D)J recombination, to progress from progenitor cells to immature B or T cells. In this review, we focus on the mechanism as it occurs in B cells, however, many aspects can be applied to T cells. Genes for the production of heavy and light chains of antigen receptors, termed variable (V), diversity (D), and joining (J), are clustered on chromosome 14 and 2/22, respectively, and require rearrangement to produce a large repertoire of functional surface receptors (Little et al., 2015; Delves and Roitt, 2000). Mechanistically, V(D)J recombination occurs in three distinct phases: recognition of recombination sites, induction of two double-strand breaks, and repair of the broken DNA by ligating the strands in a recombined configuration (Figure 1). In order to avoid off-target effects, the V(D)J recombination process is tightly regulated on a broad level by cell lineage, developmental stage, and cell cycle (Lin and Desiderio, 1994; Zhang et al., 2011; Little et al., 2015). Importantly, defects in V(D)J recombination can result in aberrant DNA joining events or loss of function, which in turn can lead to immunodeficiency and tumorigenesis, as we will describe in this review (Villa and Notarangelo, 2019).

Central to V(D)J recombination are two lymphocyte specific proteins: recombination activating genes (RAG) 1 and 2 (Teng and Schatz, 2015; Lescale and Deriano, 2017). These proteins were found to act as a tetrameric complex to mediate the cleavage phase at specific recombination signal sequences (RSSs) (Bailin et al., 1999). A multitude of these RSSs flank the V(D)J regions on the chromosome, allowing for a large variety of potential rearrangements. Chromatin remodelers and histone modifications guide the RAG proteins in their search for RSSs, as we will discuss in this review. RAG1 contains the catalytic motif essential for cleaving the DNA at the RSS, while RAG2 mediates and enhances chromosomal binding (Lescale and Deriano, 2017). Since the RAG proteins were first identified, evidence has mounted that implicates them in more than just DNA cleavage, with special roles for the complex in regulation and the hand-off to the Non-Homologous End Joining (NHEJ) repair pathway (Qui et al., 2001; Schultz et al., 2001; Lee et al., 2004). As the name indicates, NHEJ does not rely on a homologous template for repair: on the contrary, it aims to directly ligate two DNA ends together with minimal processing (Lieber, 2010; Wang et al., 2020; Zhao et al., 2020). NHEJ is the preferred pathway for V(D)J recombination, since templated Homologous Recombination would restore the original sequence, while alternative end joining pathways are too error-prone (Sallmyr and Tomkinson, 2018). Here, we will describe how the cell ensures a proper transition from the RAG-bound post-cleavage complex to the NHEJ repair complex. We also highlight some of the mechanisms the cell puts in place to make the repair phase more robust, thereby avoiding genomic instability.

In this review we aim to identify lingering gaps in the knowledge base and establish the need for continued research in the field due to the clinical implications of recombination dysfunction.

### The Fundamentals of V(D)J Recombination

#### Effective Recombination Controls Differentiation

Each antigen receptor produced through recombination in B cells will contain a heavy (IgH) and light [IgL, (Igκ or Igλ)] chain consisting of VDJ segments for IgH and VJ segments for IgL. These gene clusters extend over 3 Mb, consisting of approximately 140 Vκ, 4 Jκ, 38 Vλ, and 5 Jλ loci for use in the IgL and approximately 150 V_H_, 9 D_H_, and 4 J_H_ loci for use in the IgH (Jung et al., 2006; Ji et al., 2010; Collins and Watson, 2018).

Due to expression and degradation mechanisms of the RAG proteins discussed below V(D)J recombination is restricted to G0/G1 phase of the cell cycle. At this point the IgH locus can undergo recombination, first between D_H_-J_H_ segments before V_H_-DJ_H_ joining. Successful rearrangement of the three segments allows for production of a pre-B cell receptor (pre-BCR) and further differentiation to the small pre-B stage where IgL rearrangement can begin. Gene usage during this recombination step is skewed toward Igκ segments over Igλ (2:1 up to 95:5) (Woloschak and Krco, 1987; Lycke et al., 2015). Surface expression of the BCR in the immature B cell activates a checkpoint to determine whether the receptor is autoreactive or non-functional. If either condition occurs, secondary recombination of the Igλ gene segments is used to substitute light chains until the autoreactivity is diminished. Molecular signatures of this recombination event are the usage of more upstream V regions and more downstream J regions (Villa and Notarangelo, 2019). Following proper reactivity, the mature B cell is released from the bone marrow.

#### RAG-Mediated DNA Cleavage

The heterotetrameric recombination complex that binds the antigen receptor loci at RSSs is composed of two RAG1 subunits and two RAG2 subunits (Kim et al., 2015). Two discrete RSSs, a heptamer and a nonamer, are required for efficient binding and cleavage. Heptamer sequences follow the pattern of CACAGTG, where only the first three nucleotides are highly conserved and required for cleavage. The stronger binding nonamer sequence, ACAAAAACC, contains several conserved positions required for initial protein complex interaction. RSSs are separated by a 12 or 23 base pair spacer, which exhibits low conservation, but has the potential to introduce a significant effect on recombination efficiency (Hirokawa et al., 2020; Lee et al., 2003). Binding must occur at a pair of RSSs following the 12/23 rule, forming the paired complex (PC), which can be mediated by random collision or locus contraction (see below) (Eastman et al., 1996). Discussed later in the clinical manifestation section, cryptic RSSs (cRSSs) are common throughout the genome and, due to the sequence variation allowed by the RAG complex, may induce off-target effects. For example, frequent RAG-mediated DSBs in *c-Myc* rely only on the presence of the CAC motif of an RSS heptamer (Hu et al., 2015). Upon binding to DNA, the RAG complex induces a conformational change to the 12- and 23-RSS sites to enable efficient cleavage by RAG1. The recombination complex also utilizes high mobility group box 1/2 (HMGB1/2) to promote DNA bending, enhancing synapsis and cleavage. Once the PC is established, cleavage first occurs on a single strand via a 5’ nick at the heptamer-coding flank junction. This allows for a direct transesterification reaction where the 3’ hydroxyl group attacks the phosphate of the bottom strand. Two cleavage events in the PC generate four broken DNA ends, where two are covalently sealed coding ends (CEs) and two are blunt signal ends (SEs). This reaction takes place without a required external energy source, as the hairpin formation energy is derived from the DNA breakage. The RAG-DNA complex does not form a covalent intermediate making it distinct from other site-specific recombinases and is more similar to bacterial transposases and HIV integrase than its mammalian counterparts (Little et al., 2015). The nicking reaction can occur within minutes but the hairpinning may require hours potentially indicating simultaneous nick locations within the locus (Yu et al., 2004).

Upon cleavage the RAG complex stays associated with the broken ends forming a post-cleavage complex (PCC). This structure permits CEs to dissociate first, under the correct conditions to enter the NHEJ pathway. SEs are retained in the complex until physical disassembly can occur due to RAG2 degradation, however, this process is only speculative (Mizuta et al., 2002). Joined SEs ultimately create a non-replicative episome which is routinely lost during cell division (Smith et al., 2019). As discussed in the clinical manifestation section regulation of this component is necessary as well, due to the potential for translocation or other off-target effects if the complex is retained.

#### Non-Homologous End Joining

NHEJ proceeds through a couple of seemingly simple steps. The exposed DNA ends are first recognized by the Ku heterodimer, a ring-shaped protein (Fell and Schild-Poulter, 2015). Together with the DNA-dependent protein kinase catalytic subunit (DNA-PKcs), Ku forms the DNA-PK holoenzyme (Yue et al., 2020). This complex binds to the break site and acts as a scaffold for other repair proteins. XRCC4 (together with its binding partner Ligase IV) and XLF are recruited to the break site (Mcelhinny et al., 2000) and aid the DNA ends in coming together, a transient process called synapsis (Reid et al., 2015; Graham et al., 2017; Zhao et al., 2019). Structural studies and super-resolution microscopy have shown that XRCC4 and XLF can accomplish this by forming filaments along the DNA, which helps bridge the two ends (Hammel et al., 2010; Ropars et al., 2011; Mahaney et al., 2013; Reid et al., 2015; Chen et al., 2021). Once the DNA ends are aligned, Ligase IV seals the backbones to complete repair (Conlin et al., 2017). Over the years, it has become clear that a host of accessory factors are implicated in NHEJ, some of which are functionally redundant. We will discuss the best studied accessory factors and the implications of their functional redundancies later in this review.

If NHEJ is unavailable, repair can proceed through Alternative End Joining (alt-EJ). Alt-EJ is a less well-defined process that involves a different set of proteins, most prominently DNA Polymerase Theta (PolQ), that mediate microhomology-based annealing of resected DNA ends (Sallmyr and Tomkinson, 2018). Alt-EJ is exceptionally error-prone and usually only serves as a backup pathway. It does not typically occur during V(D)J recombination, since the hand-off of the break sites to the NHEJ machinery is tightly arranged. Indeed, deficiency in core NHEJ factors often leads to cell death (Wang et al., 2020). If certain key proteins in the hand-off fail, however, alt-EJ may be employed and lead to genome instability and disease.

With this three step process established there is still information lacking on direct influences for the recruitment of a RAG complex to RSS regions as well as subsequent pairing to the partner RSS, regulation by non-catalytic regions of RAG proteins, and certain redundant features of NHEJ factors during the repair phase, each of which will be highlighted by the following sections.

### V(D)J Regulation by RAG Non-Core Domains

**FIGURE 2:**
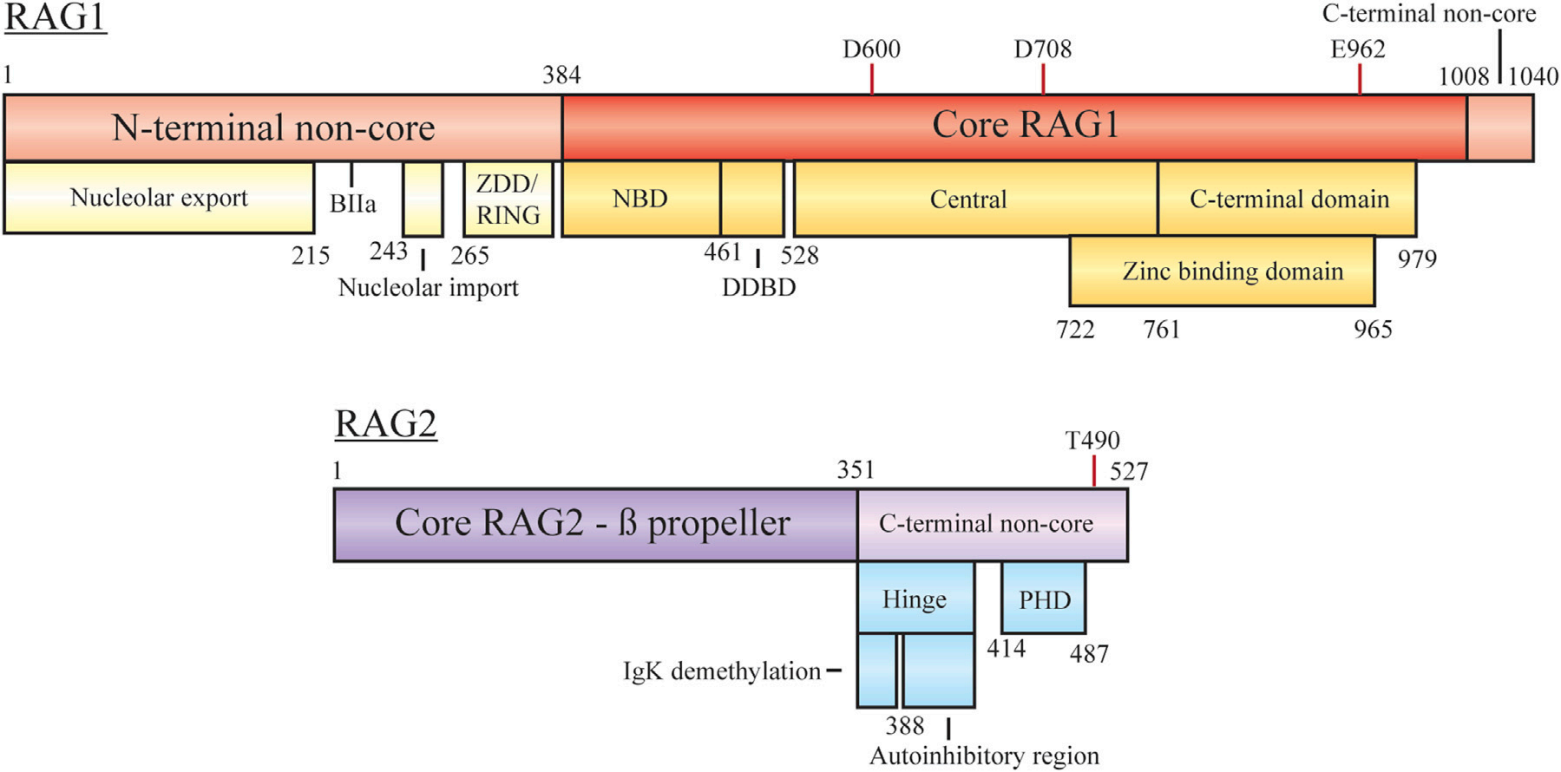
Domain organization of RAG1 and RAG2, with minimal core regions shaded darker. Subdomains/functional regions are noted below each region. Residues involved in RAG1 catalytic activity and RAG2 degradation are highlighted. Abbreviations: ZDD, zinc dimerization domain; RING, really interesting new gene; NBD, nonamer binding domain; DDBD, dimerization and DNA-binding domain; PHD, plant homeodomain.

RAG1 and RAG2 each contain various domains, where the smallest catalytically functional unit is denoted as the core region (Figure 2). These truncated constructs have been used in reconstituted functional studies due to their ease of purification. Deletion of the non-core regions allowed for recombination activity to occur, but at the cost of increased off-target effects and decreased efficiency and diversity (Talukder et al., 2004). Therefore, non-core regions are required for regulatory roles such as RSS recognition, complex stability, and handoff to repair factors. Earlier research using core proteins only and extrachromosomal substrates may require additional follow up studies to confirm physiological functions (Gigi et al., 2014).

#### RAG1 Non-Core Domain Function

RAG1 is a 1,040 aa protein consisting of a large N-terminal non-core domain (aa 1–384), the core region (384–1,008), and a short C-terminal non-core domain (1,008–1,040) (Schatz and Swanson, 2011; Little et al., 2015; Lescale and Deriano, 2017; Villa and Notarangelo, 2019). The functional core region contains the essential sites for DNA/RSS binding, homo- and hetero-dimerization and DNA cleavage (facilitated by D600, D708, and E962). The nonamer binding domain (NBD) interacts with the nonamer RSS, with the downstream dimerization and DNA binding domain (DDBD) providing a site for RAG1 homo-dimerization (Villa and Notarangelo, 2019). Regulation of contact with the heptamer RSS, ssDNA, and RAG2 is controlled by motifs within the central region. The C-terminal core region contains nonspecific DNA binding activity to mediate contact with the coding sequence flanking the RSS. A catalytic triad within the central and C-terminal domains coordinates metal ions (Mg^2+^) to activate water for ssDNA nicking activity. A zinc binding domain (ZBD) also spanning the central and C-terminal regions (722–965) is important for interaction with RAG2.

In order to understand potential interaction partners of RAG1, Brecht et al. examined association via a proximity-dependent biotin identification screening (Brecht et al., 2020). Results here indicated interaction with multiple nucleolar factors suggesting localization to this region of the nucleus outside of G1 cell cycle phase. By sequestering the protein to the nucleolus, off-target recombination events were dampened and genome stability was promoted. Upon induced recombination and G1 cell cycle arrest, RAG1 was observed to be released from the nucleolar regions and allowed to bind partner RAG2 and RSSs. Truncation of the full length protein determined that two sequences within the N-terminal non-core region were responsible for nucleolar entry (residues 243–249) and export (1–215) (Brecht et al., 2020).

In addition to nucleolar localization, the N-terminal non-core domains of RAG1 are responsible for regulation of cellular protein levels, mediation of interaction with other factors, and coordination of zinc ions, all of which act to enhance recombination activity. The zinc dimerization domain in this region (265–380) acts as a counterpart to the core ZBD, but here facilitates homo-dimerization (Lescale and Deriano, 2017). Overlapping with this domain is a RING motif, at residues 264–389, which has the capability to act as an E3 ubiquitin ligase for both autoubiquitylation and modification of other proteins (Grazini et al., 2010). RAG1 autoubiquitylation at K233 has been shown to stimulate cleavage activity in a cell free assay and exhibit post-transcriptional regulation in mice studies (Singh and Gellert, 2015; Beilinson et al., 2021). Ubiquitin modification of other proteins occurs at different stages of recombination, such as polyubiquitylation of KPNA1 or the monoubiquitylation of histones (discussed below). Sequestering RAG1 after nuclear import may be achieved by KPNA1 interaction with the basic motif BIIa within residues 218–263, only relieved by KPNA1 ubiquitylation for sub-nuclear localization (Simkus et al., 2009). However, follow up reports have discussed ubiquitylation activity mediated by additional complexes rather than the isolated RING region (discussed below) (Kassmeier et al., 2012). At the C-terminus of RAG1 the non-core region is only 32 residues, but inhibition of hairpin formation is controlled by this motif (Grundy et al., 2010). Interaction of the RAG complex with modified histones overcomes the inhibition possibly due to RAG2-mediated conformation changes associated with RAG2 C-terminal regions.

#### RAG2 Non-Core Domain Function

RAG2 is a 527 aa protein consisting of a core domain (1–351) and a C-terminal non-core domain (352–527). The core region is comprised of six Kelch-like motifs which form a six bladed ß-propeller responsible for efficient DNA cleavage (Schatz and Swanson, 2011; Little et al., 2015; Lescale and Deriano, 2017; Villa and Notarangelo, 2019). The second and sixth ß-strands are responsible for making contact with RAG1 (Little et al., 2015; Villa and Notarangelo, 2019). On its own RAG2 is monomeric, forming a 2:2 heterotetramer with RAG1 to form the RAG recombination complex (Bailin et al., 1999). As with RAG1, the non-core domain is not required for recombination activity, but regulates various parts of the recombination mechanism.

The RAG2 C-terminal non-core region is composed of two main components, an acidic hinge (351–408) and a plant homeodomain (PHD, 414–487). Although many studies have reported the RAG2 non-core region spans residues 387–527, assays with further truncations of the RAG2 core region have displayed efficient recombination with only residues 1–351 (Coussens et al., 2013; Kim et al., 2015). The acidic hinge, linking core RAG2 and the PHD, contains a high concentration of acidic residues contributing to flexibility of the region. Neutralization of the residues severely reduces the flexibility and leads to increased genomic instability as aberrant repair begins to occur (Coussens et al., 2013). Two separate regions within the acidic hinge are necessary to regulate recombination activity. Serial truncations of the hinge by Wu et al. lead to the discovery that the Igκ locus was hypermethylated upon deletion of residues 350–383 and occurred in a RAG1-independent manner (Wu et al., 2017). Demethylation of the chromosome in this context may assist in facilitating allelic exclusion, preventing further recombination on the locus. Coupled with this in the acidic hinge is an autoinihibitory function by residues 388–405, where relief is required to promote activity. As discussed below, histone recognition mediates this inhibition, with mutations bypassing the necessity for this interaction (Lu et al., 2015). The PHD component is responsible for interactions with chromatin, specifically at modified histones, based on full-length and truncated constructs submitted to ChIP-seq experiments (see below) (Teng et al., 2015). Mutations to this site, such as W453A, result in overall loss of genome localization and reduced recombination activity (Liu et al., 2007; Teng et al., 2015). At the far C-terminus phosphorylation of T490 promotes cell cycle-regulated degradation at the G1-S transition (Zhang et al., 2011). RAG2 T490A mutation can lead to persistent accumulation throughout the cell cycle as degradation is reduced. This overexpression results in continuous opportunities for RSS/cRSS target cleavage in the presence of RAG1 and recombination intermediates. The mutation also plays a role in stabilizing genomic interaction displayed by Rodgers et al. where slowed diffusion, measured in live cells via fluorescence recovery after photobleaching, was indicative of stronger interactions with modified histones (Rodgers et al., 2019).

### Recruitment of RAGs to the Site of Recombination

#### Histone Modification

To begin the process of recombination, RAG proteins must first associate with an RSS within the Mb chromosomal antigen receptor locus. The limiting of initial RAG binding can be considered a regulatory mechanism to prevent DNA nicks at random sites within the genome and may be facilitated solely by 3D diffusion to scan for sites rich in modified histones which indicate active chromatin (Lovely et al., 2020). ChIP-seq experiments by Teng et al. and Ji et al. determined the binding pattern of RAG1 and RAG2 across V(D)J segments and the entire genome revealing chromatin features which may influence the recruitment of these proteins (Ji et al., 2010; Teng et al., 2015). Within the antigen receptor loci both RAG1 and RAG2 were observed to bind at J segments in the Igκ locus and both D and J segments in the IgH locus (Ji et al., 2010). In this region, an RSS is necessary for strong binding with mutation to the nonamer sequence reducing overall recruitment. Outside of these loci, however, RAG1 localization is poorly indicated by RSS presence alone, along with cRSSs and heptamers depletion from observed binding sites suggesting that other chromatin features may play a role in RAG complex recruitment. The genomic localization of RAG2 is significantly broader with binding sites dependent on regions with high levels of methylated histone 3 (H3K4me3) and physical association determined by co-immunoprecipitation (Teng et al., 2015; Rodgers et al., 2019). As noted above, interaction of RAG2 and H3K4me3 is facilitated by the PHD of RAG2 (Matthews et al., 2007; Teng et al., 2015) The necessity of H3K4me3 binding was then determined to be due to autoinhibition of the RAG complex by RAG2 (Grundy et al., 2010). Stimulation with exogenous H3K4me3 relieved the reduced binding and catalysis, with truncation of this site uncoupling the necessity for histone recognition. Studies by Lu et al. and Bettridge et al. determined allosteric conformational changes occur to both RAG1, at the DDBD and catalytic region, and RAG2 at the autoinhibitory region allowing for increased accessibility (Lu et al., 2015; Bettridge et al., 2017). Mutations to this region can bypass the need for histone recognition to promote activity but will likely increase off-target effects (Lu et al., 2015). While RAG2 and H3K4me3 display a linear correlation of interaction, the non-linear correlation of RAG1 and H3K4me3 suggests additional features. Maman et al. used additional ChIP-seq experiments to determine possible factors for RAG1 interaction (Maman et al., 2016). H3K4me3 overlap with RAG1 was determined to be RAG2-histone dependent making it insufficient to determine RAG1 binding throughout the genome and the role methylation plays in off-target binding. Another histone modification, H3K27Ac, was instead determined to be RAG2 independent and more so influenced by N-terminal regions of RAG1, but with little direct evidence the significance is unclear (Maman et al., 2016).

#### Pairing Through Locus Contraction

**FIGURE 3:**
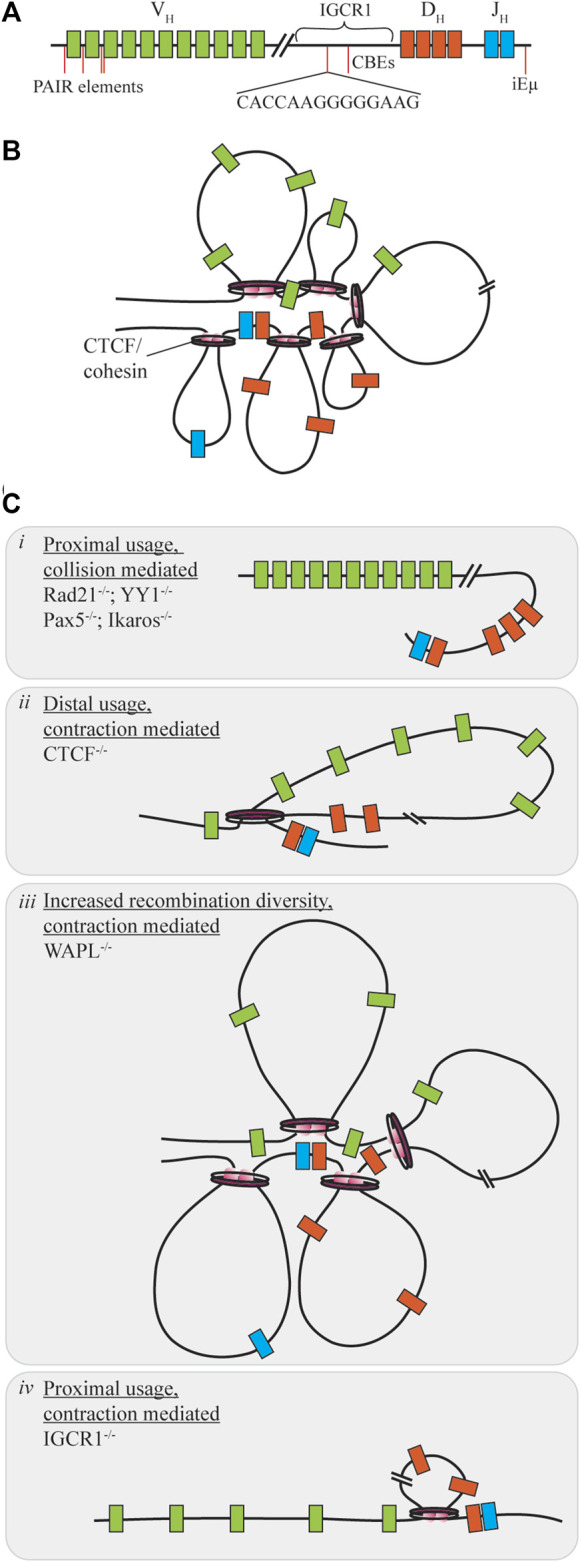
Contraction mediated conformational changes of the Ig locus. **(A)** The IgH locus contains V, D, and J segments spread over 2.8 MB of the chromosome, with regulatory regions distrubuted throughout the locus. **(B)** Under normal conditions contraction of the locus allows for gene segments to be brought into close proximity as the rosette conformation is formed with varying loop sizes. **(C)** When proteins mediating this contraction are dysregulated V**(D)**J recombination diversity may be skewed towards one segment of the V_H_ region through collision (i) or contraction (ii, iv). Only under WAPL repression is diversity increased as cohesin-mediated loop size increases due to cohesin retention on chromatin (iii).

In addition to initial binding, the RAG-DNA complex must associate with a second, partner RSS to perform recombination. Regulation at this point is achieved through the physical proximity of the V gene segments, which are spread across over 2 Mb of DNA (Figure 3A). While proximal segments may be paired via random collision, large scale chromosomal conformational changes are utilized to direct pairing at both central and distal regions within the loci, skewing interaction partners and providing a diverse set of antigen receptors (Figure 3B). Various mechanisms of chromosomal looping enables regions to be brought into close proximity during pro- and pre-B stages via proteins such as YY1, Ikaros, Pax5 and CTCF.

Distal V_H_ utilization is facilitated by each of the proteins under slightly different mechanisms. Within the IgH locus are enhancer regions, intronic enhancer (iEµ) and a 3’ regulatory region which provide sites for contraction protein binding. Yin Yang 1 (YY1) is a zinc finger protein with multiple functions in regards to transcription activation and repression (Liu et al., 2007). Knockouts of this protein during B-cell development yield a block at the pro-B cell stage due to insufficient V_H_-DJ_H_ recombination without influencing expression of additional V(D)J recombination components (Figure 3Ci). Using ChIP and 3D DNA FISH Liu et al. were able to determine that YY1 binds to iEµ sites to provide a node for locus contraction (Liu et al., 2007). Deletion of iEµ does not inhibit YY1 binding to the overall chromosome loci, indicating that additional sequences and factors influence the contraction (Guo et al., 2011). In addition, YY1 may have heterotypic interactions with CTCF, however, changes to rearrangement if CTCF levels are decreased are not as pronounced as the knockdown of YY1 in reducing recombination efficiency (Degner et al., 2011; Weintraub et al., 2017). Ikaros, also a zinc finger protein, contributes various roles in differentiation control and chromosome accessibility (Reynaud et al., 2008). In a similar manner, reduction of Ikaros leads to overall low V_H_-DJ_H_ recombination with heavily skewed usage of proximal V_H_ segments (Figure 3Ci). Pax5, a B-cell commitment factor for differentiation, controls transcription, but also functions by positioning chromatin towards the central nuclear regions ensuring active chromatin is in an extended state and promotes locus contraction (Ebert et al., 2011; Fuxa et al., 2004). When Pax5 is deleted, cells will have characteristic features of uncommitted progenitors, such as the ability to change differentiation pathway following cytokine stimulation, and exhibit reduced diversity due to a 50-fold reduction of distal V recombination (Figure 3Ci). Contraction by Pax5 is mediated through Pax5-activated intergenic repeats (PAIRs) over 750 kB of the distal V_H_ gene which allow for interaction with iEµ sites (Verma-Gaur et al., 2012). *Via* Bio-ChIP-chip Ebert et al. showed that this mechanism is specifically lost in the pre-B cell stage where other mechanisms must be used to promote distal gene usage (Ebert et al., 2011).

CCCTC-binding factor (CTCF) is another zinc finger protein with diverse functions for transcriptional control, but also mediating chromosomal contacts across the genome (Phillips and Corces, 2009; Degner et al., 2011). Deletion of CTCF reduces overall B-cell maturation and arrests development at pre-B stage for those progressing that far. Mediation of contraction here is regulated by the presence of CTCF-binding elements (CBEs), 14 bp conserved targets within the Ig loci (Hu et al., 2015). Association of pairs of convergently oriented CBEs bound by CTCF and the cohesin complex form loop domains of the antigen receptor loci restricting the V segments (Zhao et al., 2016; Zhang et al., 2019). Cohesin, consisting of multiple subunits including Rad21, will form loops of various sizes by extrusion of chromatin in an ATP-dependent manner until reaching a CTCF bound CBE (Ba et al., 2020). The loop extrusion process is dynamic and allows for a subset of CTCF/cohesin formed loops to exist at any given time enhancing genetic diversity (Degner et al., 2011). Large chromosomal structural rearrangements also compete with the short range collisional recombination which is extinguished during CTCF downregulation as RAG proceeds to distal V_H_ regions without obstruction therefore limiting diversity (Figure 3Cii), yet in the Igκ locus proximal usage is increased (De Almeida et al., 2011; Ba et al., 2020; Zhang et al., 2022). In contrast, deletion of cohesin subunit Rad21 eliminates all recombination at most sites, except for proximal regions which form a synapse due to collisional diffusion (Figure 3Ci). Cohesin unloading is regulated by the expression of WAPL throughout the cell cycle. Pax5 repression of the WAPL promoter during pro- and pre-B cell stages allows for increased cohesin residence time on chromatin extending loop sizes by circumventing CBE obstructions (Hill et al., 2020; Dai et al., 2021). Under these circumstances, there is a general increase of recombination at all sites (Figure 3Ciii). For a comprehensive look into cohesin-mediated loop extrusion, we refer the reader to the recent review by Zhang et al., (2022).

Enhancer and intergenic regions of the antigen receptor loci are also important for CTCF mediated loop extrusion as deletion or mutation of these sites provides limited diversity and dysfunction. iEµ in the IgH locus is required for efficient recombination, where deletion increases proximal gene usage and reduced chromosomal relocation to the central nuclear regions to limit total accessibility (Guo et al., 2011). The intergenic control region 1 (IGCR1), between V_H_ and D_H_ segments, contains CBEs to suppress V_H_ usage prior to D_H_-J_H_ rearrangement. Deletion of the IGCR1 region promotes proximal V_H_ usage, but also induces off-target breaks spreading up to 120 kb upstream of the proximal V_H_ segments for potential cRSS replacement (Figure 3Civ). CBEs within the V_H_ region prevent distal usage past these segments in the absence of IGCR1 regulation (Hu et al., 2015). The Vκ-Jκ locus contains additional DNase hypersensitive (HS) regions which influence chromosomal conformation change. Upon deletion of HS3-6 there is only a moderate decrease of middle gene usage, with overall insignificant changes to locus contraction. However, HS1-2 deletion results in at least a 7-fold increase of proximal gene usage with 3D DNA FISH indicating a 50% decreasing in overall contraction of the Ig locus (Xiang et al., 2013).

### Hand-Off of the Post-Cleavage Complex to the DNA Repair Machinery

Cleavage by the RAG proteins triggers the DNA damage response. A proper hand-off to NHEJ machinery is essential for successful recombination: alt-EJ may lead to aberrant joining events and genomic instability, especially in p53-deficient environments. The idea that the role of the RAG proteins extends beyond the cleavage step, came when several RAG mutants were found to be proficient for cleavage but exhibited aberrant joining (Qui et al., 2001; Schultz et al., 2001; Lee et al., 2004). Exactly how the RAG proteins channel the repair to NHEJ is unclear, although three elements seem to be important for pathway choice. The first is the dependence of RAG activity on the cell cycle: by limiting recombination to G1, HR is not available (Zhang et al., 2011). Moreover, expression of PolQ is very low in G0 and G1, limiting the possibilities for alt-EJ as well (Yu et al., 2020). RAG2 residue T490 is a CDK phosphorylation site, that is, instrumental in targeting RAG2 for breakdown when the cell moves to S phase; indeed, the T490A mutation is enough to lead to aberrant recombination (Zhang et al., 2011). The second element is the ability of the RAG1 N-terminal domain to bind a multi-protein complex, containing Ku, that steers repair to NHEJ (Raval et al., 2008; Kassmeier et al., 2012). Ku was recently shown to suppress alt-EJ of RAG-induced DSBs, indicating it aids in shepherding breaks to NHEJ during V(D)J recombination (Liang et al., 2021). Other proteins in the complex are VprBP, DDB, Cul4A and RocI: these act as a RING E3 ligase that can ubiquitylate nearby proteins (Kassmeier et al., 2012). Disruption of VprBP (by conditional excision of two exons) leads to defects in recombination and increased mutations in the D and J segments in mice. Based on the mutational signature, the authors suggest that VprBP specifically regulates terminal transferase activity through a mechanism that involves ubiquitylation of an unknown target, and thus suppresses other error-prone repair pathways. The third element is the stability of the PCC, which was found early on to influence the choice of repair pathway: unstable PCCs are more prone to lead to alt-EJ instead (Lee et al., 2004). Stability of the PCC seems to be closely related to the conformation of the acidic hinge in the RAG2 C-terminus, an intrinsically disordered domain with a high negative charge. Mutations that neutralize this charge destabilize the PCC and allow repair through alt-EJ (Coussens et al., 2013). The RAG2 C-terminus has been shown to influence pathway choice on more occasions (Corneo et al., 2007; Gigi et al., 2014; Mijuskovic et al., 2015). The exact mechanism, however, still remains unclear. Interestingly, the RAG2 C-terminus was found to be redundant with XLF for what appears to be a function in stabilization of DNA ends: mice that are deficient for XLF but express the core RAG2 show severe defects in V(D)J recombination which in turn leads to lower numbers of lymphocytes (Lescale et al., 2016a). This opens the intriguing possibility that the RAG proteins interact with XLF in the synaptic complex.

**FIGURE 4:**
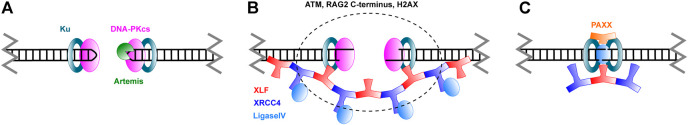
Schematic overview of Non-Homologous End Joining. **(A)** Broken DNA ends are recognized by Ku and DNA-PKcs. Hairpins in coding ends are opened by the Artemis nuclease. **(B)** Ends are brought in close proximity in a process called synapsis. XLF and XRCC4 are thought to form filaments that mediate this process. As discussed in the text, ATM, the RAG2 C-terminus and H2AX also have a role in synapsis. **(C)** Ends are ligated by Ligase IV. PAXX joins in this stage of repair. Please note that, while care was taken to represent the architecture of the complexes as accurately as possible, many structural features still remain unknown.

Although both proceed through NHEJ, the repair of coding and signal ends is slightly different. Signal ends are blunt (Roth et al., 1993), while coding ends are hairpins that need processing (Roth et al., 1992). After cleavage, the RAG proteins are more likely to stay bound to the signal ends, at least *in vitro* (Ramsden and Gellert, 1995; Livak and Schatz, 1996; Agrawal and Schatz, 1997; Hiom and Gellert, 1998). The sealed coding ends, which will be quickly bound by Ku, are a target for DNA-PKcs (Figure 4A). Once this enzyme binds to the break site, it will act as a regulator for further processing steps. As has recently been shown through a crystal structure of DNA-PKcs, the hairpin DNA substrate will trigger DNA-PKcs to phosphorylate itself, which results in a large conformational change that creates room for the Artemis endonuclease to bind (Liu et al., 2022). Artemis is capable of opening the hairpins, which it does asymmetrically to create a 3' 2 nucleotide overhang (Ma et al., 2002; Karim et al., 2020; Yosaatmadja et al., 2021). This overhang is the reason repaired coding ends typically show indels; it serves as a substrate for the TdT polymerase, which can add nucleotides to the overhang without the need for a template (Motea and Berdis, 2010). As such, 3’ overhang elongation is an additional mechanism to create diversity at V(D)J junctions. The two ends, which may have diffused apart in the meantime, then need to be brought together in a synaptic complex for repair to proceed. The signal ends, on the contrary, are blunt and held together by the RAG proteins, obviating the need for a pre-processing step or formation of a synaptic complex. As a consequence, the aforementioned interaction between XRCC4 and XLF to form filaments that bridge DNA ends is not necessary for signal end repair (Roy et al., 2012). Signal end repair does, however, need kinase activity from either DNA-PKcs or ATM, probably to remove the RAG proteins from the break site (Zha et al., 2011b; Gapud et al., 2011; Gapud and Sleckman, 2011). In the absence of filament formation, signal end repair is also more dependent on XRCC4 than on XLF, which is in line with the role of XRCC4 to carry Ligase IV to the break site.

### Functional Redundancies and Newly Identified Non-Homologous End Joining Factors

The core factors Ku, XRCC4 and Ligase IV are absolutely essential for NHEJ: knock-outs of these genes in mice lead to severe phenotypes or embryonic lethality (reviewed in Wang et al., 2020 and Zhao et al., 2020) (Wang et al., 2020; Zhao et al., 2020). There is, however, a considerable degree of functional redundancy among most other NHEJ factors. These redundancies make repair more robust, and prevent genomic instability associated with unrepaired breaks or alt-EJ pathways (Chang et al., 2017). A number of functional redundancies have been identified in mouse models: while a single knock-out of a redundant NHEJ factor may only lead to a mild phenotype, a more severe phenotype in a double knock-out suggests a functional redundancy between those two NHEJ factors. These redundancies have for years obscured the role some proteins play in NHEJ, like XLF (Li et al., 2008) or the more recently identified roles of PAXX (Ochi et al., 2015; Xing et al., 2015) and MRI (Hung et al., 2018). For this reason, functional redundancies with XLF have been particularly well studied and have shed some light on the molecular mechanism of end joining. For a relatively recent overview of the effect of single or double knock-outs in NHEJ we would like to refer to Wang et al., (2020). Here, we focus on redundancies of XLF with some of the newly identified NHEJ factors PAXX and MRI, and with ATM and H2AX.

PAXX, a paralog of XRCC4 and XLF, was discovered not so long ago as a player in NHEJ (Ochi et al., 2015; Xing et al., 2015). PAXX bears strong structural similarity to XRCC4 and XLF, but is slightly smaller. Consistent with a role in DNA repair, PAXX is recruited to damage sites; moreover, PAXX deficiency leads to an increased sensitivity to ionizing radiation in human somatic U2OS cells (Ochi et al., 2015). The conserved C-terminal region of PAXX binds to the N-terminal region of Ku80, revealing a mechanism for PAXX recruitment to DSBs (Ochi et al., 2015; Liu et al., 2017). This interaction is essential, since PAXX does not appear to have any DNA binding activity by itself. Considering its similarity to XRCC4 and XLF, it was surprising to find that PAXX does not participate in bridging of DNA ends. Rather, its interaction with Ku seems to promote the accumulation of XLF and Polymerase Lambda at DSBs (Craxton et al., 2018), as well as to promote further accumulation of Ku (Liu et al., 2017). In the context of simple DSBs, PAXX function seems to be redundant with XLF, whereas PAXX and XLF work together in the repair of more complex breaks (Xing et al., 2015). Interestingly, PAXX is dispensable for V(D)J recombination in a mouse pro-B cell line, as long as XLF is present (Kumar et al., 2016; Lescale et al., 2016b). This reveals a functional redundancy between these two proteins in the context of V(D)J recombination. Since XLF itself is redundant with ATM in the same context, one might wonder if PAXX, in turn, is also redundant with ATM. It turns out this is not the case, indicating that these proteins act at more than one stage and only some functions overlap (Kumar et al., 2016). Lescale et al. proposed a two-tier model of an initial synapsis stage and a subsequent ligation stage (Lescale et al., 2016b). In the synapsis stage, XLF forms filaments with XRCC4, bridging the break site (Figure 4B). ATM has a similar, but independent role. In the ligation stage, XLF stabilizes the ligation complex. Here PAXX has a similar function, thus creating the redundancy with XLF (Figure 4C). Gaps due to incompatible ends can then be filled in by Polymerase Lambda. In line with this redundancy, mouse models showed that PAXX is dispensable for normal development (Gago-Fuentes et al., 2018), but PAXX and XLF double knock-out mice died as embryos (Balmus et al., 2016; Liu et al., 2017; Abramowski et al., 2018). In summary, the role of PAXX in NHEJ fits with the general theme of redundancy.

Another recently identified player in NHEJ is the Modulator of Retroviral Infection (MRI). This small disordered protein interacts with DNA-PKcs, Ku, PAXX, XLF and XRCC4 through its N-terminal domain and with ATM and the MRN complex through its C-terminus (Arnoult et al., 2017; Hung et al., 2018). MRI is thought to stabilize these other proteins on the chromatin around the break site, potentially by forming multimeric structures through its disordered regions (Hung et al., 2018). In mice, MRI deficiency alone does not result in a detectable phenotype. However, *MRI*-/- *XLF*-/- and *MRI*-/- *DNA-PKcs*-/- mice show embryonic lethality, while the double knock-out *MRI*-/- *PAXX*-/- does not result in a severe phenotype (Castaneda-Zegarra et al., 2020). This again indicates a degree of redundancy between different repair factors; the severe phenotype with DNA-PKcs and the milder phenotype with PAXX suggests that the major role of MRI is relatively early in repair, during the synapsis stage.

Interestingly, XLF is also functionally redundant with ATM (Zha et al., 2011a; Xing and Oksenych, 2019). The ATM kinase is an important regulator in NHEJ and the DNA damage response in general (Lee and Paull, 2021). It phosphorylates H2AX, which alters the local chromatin architecture to create a favorable environment for DNA repair processes. XLF also has redundant functions with H2AX directly. Consistent with all of this, the XLF/ATM redundancy only exists in the context of chromatin, and does not occur in assays that utilize extrachromosomal DNA (Zha et al., 2011a). It has been shown that H2AX keeps break sites together (Yin et al., 2009), and is therefore likely to have a role in the synapsis phase of repair, where the redundancy with XLF would then originate. The exact molecular mechanism, however, remains unclear. Rather than interacting directly with the coding and signal ends, phosphorylated H2AX could keep the double-strand breaks together in a confined space by forming a biomolecular condensate in the chromatin. The role of such condensates or repair foci has received a lot of attention recently [reviewed in (Fijen and Rothenberg, 2021)]. As discussed earlier, chromatin remodeling is also a key process in the initiation of recombination. Further research into the role of the chromatin architecture and biomolecular condensates throughout the recombination process could provide an interesting new perspective on the regulation and efficiency of V(D)J recombination.

### Clinical Manifestation of V(D)J Recombination Defects

While healthy cells should be able to restrict recombination activity to G1 cell cycle phase, isolating RAG-mediated breaks to prevent off-target repair pathways, the large number of components involved during this mechanism can lead to harmful implications. Various types of immunodeficiency and potential tumorigenesis can be initiated by aberrant translocations and deletions through RAG complex mutation or deficiency.

#### Immunodeficiency

Deficiency in RAG proteins results in an overall lack of recombination efficiency and diversity, with lower expression leading to a harsher clinical outcome. This deficiency can lead to several phenotypes including severe combined immunodeficiency (SCID), combined immunodeficiency with granulomas or autoimmunity (CID-G/AI) and Omenn Syndrome (OS) (Schwarz et al., 1996; Schuetz et al., 2008). For an extensive analysis of the pathogenesis of these RAG-mediated deficiencies we refer the reader to the recent review from Bosticardo et al. (Bosticardo et al., 2021). SCID, and to the lesser extent CID-G/AI, can cause major vulnerability to minor infections, with current treatment through methods such as bone marrow transplant (Buckley, 2004). Recent large cohort studies for RAG deficiency show occurrence in 12% of SCID cases and 42% of atypical SCID cases (Dvorak et al., 2019). OS patients display a complex pathogenesis with symptoms similar to SCID, except an estimated 90% of cases are due to RAG mutations (Marrella et al., 2011). Over 60 naturally occurring mutations resulting in immunodeficiency have been mapped to just the core regions of RAGs with effects such as destabilized structures between RAGs or other components, decreased DNA binding, and catalytic deficiency (Kim et al., 2015). Two example mutations related to OS, V779M and C328G in RAG1, reduce recombination through different mechanisms, decreased cleavage efficiency and joint formation, respectively (Grazini et al., 2010; Matthews et al., 2015). Lee et al. and Tirosh et al. determined the recombination efficiency of RAG1 and RAG2, respectively, using mutations present in patient samples with varying disease type and severity (Lee et al., 2014; Tirosh et al., 2019). A high density of mutations occur in the NBD and mutations to this region or the heptamer binding motif of RAG1 tend to exhibit significantly lower activity even though the protein is catalytically active (Lee et al., 2014). In RAG2 samples, the overrepresentation occurs in the PHD, affecting histone interaction and the autoinhibitory mechanism. Various mutations in RAGs may also circumvent the checkpoints related to autoreactivity leading to reduced functional circulating B-cells in addition to the reduced repertoire (Villa and Notarangelo, 2019). As noted in Figure 3, interfering with locus contraction leads to decreased antigen receptor diversity and mutations to proteins involved, such as cohesin subunits and Ikaros, have been associated with immune disease (Bjorkman et al., 2018; Kuehn et al., 2021). Recurrence of the same low activity mutations in RAGs or other proteins required for recombination could allow for prediction of disease severity in newly diagnosed patients and potential for personalized medicine for achieving a significant level of recombination based on genotype.

#### Tumorigenesis

Human lymphomas can involve RAG-mediated deletions or potential translocations between the Ig locus V(D)J segments and non-Ig locus. The main area of concern is the presence of cRSSs which mimic the RSS motif, but exist outside of the antigen receptor loci (Onozawa and Aplan, 2012; Hu et al., 2015; Teng et al., 2015). RAG-mediated cleavage at cRSS sites could be detrimental to cell viability as uncontrolled regions are disturbed. Notch-1, a ligand activator transcription factor which transduces signaling information from the cell surface to the nucleus, contains 14 cRSSs within the 30 kb locus (Mijuskovic et al., 2015). N-terminal truncation caused by cRSS-mediated deletion exhibits constitutive ligand independent intracellular activity. Using ChIP-seq data from Ji et al., RAG2-H3K4me-Notch-1 5’ binding and colocalization indicates that RAG-mediated cleavage has a high likelihood of occurring in this region (Onozawa and Aplan, 2012). Multiple sets of cRSSs are also involved in the deletion of Jak1 exons 6–8, leading to activation with multiple roles in cell growth and survival (Mijuskovic et al., 2015). Additional RAG-mediated deletions have occurred at Trat1, Phlda1, Agpat9, CDKN2a/b, Ikaros, and have been attributed to *Tal1-Sil* fusion (Mullighan et al., 2008; Onozawa and Aplan, 2012; Larmonie et al., 2013; Mijuskovic et al., 2015). Even so, a majority of other oncogenic breakpoints detected in lymphomas do not contain cRSS sites and may be due to event at non-B form DNA structures (Raghavan et al., 2004).

Translocation due to off-target RAG-mediated events would be more detrimental to cell viability, but have eluded direct detection in the genome. Translocations themselves are common and likely due to recombination events, but as of July 2019, there have been no documented cases of leukemia and lymphomas which could be traced directly to a RAG-mediated transposition event (Zhang et al., 2017; Smith et al., 2019). Even translocations which involve the antigen receptor loci, such as *Bcl2-IgH* or *BCR-ABL1*, lack substantial evidence of initial RAG-mediated DSBs (Mossadegh-Keller et al., 2021; Yuan et al., 2021). This may be due to a lack of ability to screen for these type of lesions as the limitations of some sequencing methods may overlook certain breakpoint features, however, recent improvements to next generation sequencing and whole genome sequencing will allow for higher discovery rate of these off-target RAG induced breaks (Nordlund et al., 2020; Afkhami et al., 2021; Xiao et al., 2021). The excised signal circle (ESC) complex consisting of the SEs, RAG proteins, and other factors is another source of potential reintegration into the genome (Kirkham et al., 2019). This complex can be extremely dangerous for oncogenic upregulation due to the presence of V region adjacent promoters. More likely are asymmetric cleavage events (“cut and run”), where a closed ESC binds and cleaves at a cRSS before continuing on a series of unchaperoned DNA DSBs. These events have yet to proven *in vivo*, yet acute lymphoblastic leukemia patients have shown oncogenic activation through translocation events, such as *ETV6-RUNX1* gene fusion, which could be facilitated by RAG-mediated ‘cut and run’ events, however, more research is necessary to understand a direct involvement of RAGs in this type of tumorigenesis (Papaemmanuil et al., 2014; Kirkham et al., 2019). In addition, translocation of the DNA fragment of the ESC complex may not be due to new RAG-mediated cleavage events, but instead insertion at independently formed DSBs, leading to further genomic instability (Antoszewska-Smith et al., 2017; Rommel et al., 2017).

## Concluding Remarks and Outlook

Historically, focus on V(D)J recombination research has been on the molecular mechanism of single recombination events, while more recently the regulation has gained more attention. Efficient V(D)J recombination is dependent on a tight regulation of locus recognition, DNA cleavage and repair. Here we discussed the latest insights regarding target binding and robustness of the NHEJ pathway, but details of some key processes remain to be established. RAG non-core domains have been only recently studied for their regulatory roles, noted here is the importance of these regions in sub-nuclear localization (PHD), efficient transition to repair (acidic hinge), and maintenance of protein degradation (RING). Additional research will be necessary to further investigate these roles and potential allosteric mechanisms influencing function. We discussed RSS binding and pairing, but Maman et al. determine histone modification itself is not enough for initial recruitment (Maman et al., 2016). Simple 3D diffusion may account for RSS association, however, the choice of a partner RSS may be influenced by several rounds of binding/release during locus contraction. The role of local chromatin architecture and condensate formation has been gaining significant traction lately, with the role of disordered protein domains and long non-coding RNAs being recognized (Fijen and Rothenberg, 2021). We see potential for advanced imaging techniques to resolve the recruitment dynamics and large-scale features of the recombination center and repair foci. We noted here that the repair-associated kinases ATM and DNA-PKcs are required for efficient recombination, but the specific contribution of each and potential redundant roles remain poorly understood. We anticipate that the phosphorylation profile of repair factors has an impact on stability of the recombination complex, joint formation, and repair factor recruitment. Dysregulation of these events have significant influence on off-target breaks or repair deficiency resulting in immunocompromising phenotypes and potential tumorigenesis. Certain mutations and RAG-mediated deletions are implicated in these disease states yet likely direct involvement of RAGs in oncogenic translocations fails to be detected. Extraction of this aberrant joining from the tumor genome proves challenging, but would be vital for clinical therapeutics and personalized medicine.
